# Hydroalcoholic extract of *Caryocar brasiliense* Cambess. leaves affect the development of *Aedes aegypti* mosquitoes

**DOI:** 10.1590/0037-8682-0176-2020

**Published:** 2020-09-11

**Authors:** Hevilem Letícia Moura do Nascimento Morais, Talita Carneiro Feitosa, João Gustavo Mendes Rodrigues, Maria Gabriela Sampaio Lira, Ranielly Araújo Nogueira, Tássio Rômulo Silva Araújo Luz, Nêuton Silva-Souza, Nerilson Marques Lima, Teresinha de Jesus Aguiar dos Santos Andrade, Guilherme Silva Miranda

**Affiliations:** 1 Instituto Federal de Educação, Ciência e Tecnologia do Maranhão, Departamento de Educação, São Raimundo das Mangabeiras, MA, Brasil.; 2 Universidade Estadual do Maranhão, Departamento de Química e Biologia, São Luís, MA, Brasil.; 3 Universidade Federal de Minas Gerais, Departamento de Parasitologia, Belo Horizonte, MG, Brasil.; 4 Universidade Federal do Maranhão, Departamento de Patologia, São Luís, MA, Brasil.; 5 Universidade Federal do Maranhão, Departamento de Farmácia, São Luís, MA, Brasil.; 6 Universidade Federal de Juiz de Fora, Departamento de Química, Juiz de Fora, MG, Brasil.

**Keywords:** Emergence inhibition of mosquitoes, Flavonoids, Plant extracts, Vectors

## Abstract

**INTRODUCTION:**

Curtailing the development of the aquatic immature stages of *Aedes aegypti* is one of the main measures to limit their spread and the diseases transmitted by them. The use of plant extracts is a promising approach in the development of natural insecticides. Thus, this research aimed to characterize the inhibitory effect of the hydroalcoholic extract of *Caryocar brasiliense* leaves on the emergence of adult *A. aegypti* and the main substances that constitute this extract.

**METHODS:**

*C. brasiliense* leaf extract was prepared by ethanol (70%) extraction. Bioassays using L3 larvae were performed at concentrations of 200, 300, 400, and 500 ppm. We identified the major secondary metabolites present in this extract, and performed toxicity tests on an off-target organism, *Danio rerio*.

**RESULTS:**

We observed a significant delay in the development of *A. aegypti* larvae mainly at a concentration of 500 ppm, and estimated an emergence inhibition for 50% of the population of 150 ppm. Moreover, the *C. brasiliense* leaf extracts exhibited low toxicity in *D. rerio*. The main compounds found in the extract were quercetin, violaxanthin, myricetin3-O-hexoside, methyl-elagic-3-arabinose acid, and isoquercitrin.

**CONCLUSIONS:**

Herein, we demonstrate the inhibition of mosquito development by the hydroalcoholic extract of *C. brasiliense* and suggest substances that may act as active principles.

## INTRODUCTION

The mosquito *Aedes aegypti* L. (Diptera: Culicidae) is the main vector for several serious mosquito-borne infectious pathogens, such as Dengue, Yellow fever, Chikungunya, and Zika viruses, in many parts of the world^1^ ^,^ ^2^. Almost half of the world’s population is now at risk of these diseases^3^. The worldwide distribution and the difficulty in controlling *A. aegypti* can be attributed to their biological characteristics, such as their adaptability to anthropogenic conditions and preference for human blood over other vertebrates^4^, gonotrophic disagreement (the ability to have a blood meal for each batch of eggs produced), and the resistance of their eggs to desiccation for months^5^ ^,^ ^6^. 

While there is no effective vaccine yet for Zika and Chikungunya, and the vaccination tests for Dengue remain without satisfactory results, the main methods to combat these arboviruses are focused on the immature aquatic forms of the mosquito vector. The use of synthetic chemical compounds for this purpose poses disadvantages as most of them are non-biodegradable, and are toxic to off-target organisms. In addition, the emergence of populations resistant to major insecticides has been reported^7^ ^,^ ^8^.

A promising alternative is the use of natural compounds with insecticidal effects, as these substances generally do not harm the environment, and exhibit low toxicity to off-target organisms^9^ ^,^ ^10^. As plants produce many chemical compounds during metabolism (secondary metabolites), extracts and essential oils from different plant structures are promising alternatives for the sustainable combat of *A. aegypti* ^11^ ^,^ ^12^. 

The “Pequizeiro” (*Caryocar brasiliense* Cambess.) plant species found in the Brazilian Cerrado biome, is part of the Caryocaraceae family, which constitutes about 16 species (12 found in the Brazilian territory)^13^. The fruit of *C. brasiliense* has a huge economic potential, especially in the Brazilian food culture, where it is used in different traditional dishes. Herbal medicines produced from its flowers and leaves are used in several treatments. Numerous studies have demonstrated the important pharmacological benefits of several secondary metabolites from *C. brasiliense* leaves, including flavonoids (anti-inflammatory, antiallergic, antiulcerogenic, and antiviral), tannins (anti-inflammatory), coumarins (antimicrobial, antiviral, anti-inflammatory, and antitumoral), and saponins (anti-inflammatory, larvicidal, expectorante, and molluscicidal)^14^.

However, a detailed chemical characterization and the biological effects of the hydroalcoholic extract of *C. brasiliense* leaves on the development of the aquatic immature stages of *A. aegypti*, have not been evaluated, which is the main objective of this study.

## METHODS

### Collection of plants and preparation of the hydroalcoholic extract

The leaves of *C. brasiliense* were collected from Sucupira do Norte, Maranhão state, Brazil (6°28′43″S 44°11′20″W) in the morning. The plant was identified at the Rosa Mochel Herbarium of the State University of Maranhão (UEMA), with specimen voucher number 5515. The plant material was collected, cleaned, dried, and powdered. The powder was macerated with a 70% hydroalcoholic solution and mixed every 12 hours, for a total of 48 hours, at a hydromodule of 1:5 (w/v). The extract was first filtered five times, and then filtered and concentrated under reduced pressure to obtain a dry extract^15^. The final yield was 10% of the weight of the crushed dry leaves. 

### Test organism

A population of *A. aegypti* was initially isolated from the field in 2017 (São Raimundo das Mangabeiras city, Brazil) using ovitraps and maintained in the laboratory at 28 ± 1 °C and 70 ± 5% relative humidity at a photoperiod of 14:10 (light/dark). The larvae were obtained from the mosquito eggs and hatched by submersion in distilled water. The larvae were reared in plastic basins and fed with pulverized cat food (0.25 mg/larva/day). 

### Insect growth regulators bioassay

This assay was performed according to World Health Organization^16^ guidelines, with some modifications. Briefly, 30 third instar larvae (L3) were exposed to various concentrations of the leaf extract in 500 mL distilled water (200-500 ppm). The control group with the same number of larvae were exposed only to distilled water (500 mL). A small amount of pulverized cat food at a concentration of 10 mg/L was provided every second day. The number of larvae, pupae, and adults was counted and compared between each group at intervals of 24-120 hours. Mortality, deformities, or morphogenetic effects in the adult mosquitoes were also recorded at the same time intervals. The experiment was carried out in 3 glass beakers for each concentration (technical triplicate), repeated twice on different days (biological repetitions). The effects are expressed as percentages of IE (inhibition of emergence) based on the number of larvae that do not successfully develop into viable adults in the experimental groups (T) compared to the control group (C), using the following formula:

$$\text{IE}\left(\text{\%}\right)\;\text{=}\;\text{100 -}\left(\frac{\text{T x 100}}{\text{C}}\right)$$

### Toxicity on off-target organisms

To investigate the toxicity of the extract on other organisms and to ensure environmental safety, we performed tests with adult *Danio rerio* (zebrafish) specimens according to the methodology described in the Brazilian Association of Technical Standards^17^. Briefly, groups of 4 zebrafish were placed in glass containers with solutions containing the extract of *C. brasiliense* leaves. The effect of exposure to the test solutions for 48 hours at concentrations between 75-250 ppm from the IE_50_ (*C. brasiliense* leaves extract) was evaluated in three glass containers for each concentration (technical triplicate) and repeated four times on different days (biological repetitions). For every 24 hours, the variables such as pH, conductivity, dissolved oxygen, and temperature were observed without replacing the solutions, and the dead animals were counted. The negative control group was kept in a chlorinated water solution, and the positive control group in a potassium dichromate solution (K_2_Cr_2_O_7_). The experimental protocol was approved by the Animal Use Ethics Committee (CEUA) of the Federal University of Maranhão (UFMA) under the registration number 23115.009327/2017-10.

### Screening of phytochemical compounds

High-resolution electrospray ionization mass spectra (HRESIMS) data in positive ionization mode was obtained using an LTQ Orbitrap XL Hybrid Fourier transform mass spectrometer discovery system (Thermo Scientific Instruments), coupled to a Thermo Instruments HPLC system (Accela PDA detector, Accela autosampler and Accela pump, Thermo Scientific Instruments). The following conditions were used: 4.5 kV capillary voltage, 260 ºC capillary temperature, 10-20 arbitrary units auxiliary gas flow rate, 40-50 arbitrary units sheath gas flow rate, 4.5 kV spray voltage, and 100-1000 amu (maximum resolution 30.000) mass range. The software used for the acquisition and processing of spectrometry data was Xcalibur (Thermo Scientific^®^).

Extract screening was performed using a Shimadzu LC-10AD high performance liquid chromatograph (analytical, binary) with a Shimadzu SIL-10A autoinjector coupled to a diode array detector with a scanning range of 195-650 nm, and a minimum step size of 1 nm. HPLC separations were performed using a Sunfire 150 x 4.6 mm C18 column (Waters) at a flow rate of 1.0 mL/min, injection volume of 50 µL, and 200-600 nm UV. To obtain the profile in HPLC-PAD and LC/ESI-MS, a solution of 1 mg/mL of the sample in 95% methanol was prepared, and then Teflon membrane filtration (0.45 µm) was performed. Analysis with solvent systems A (water) and B (methanol with 0.1% formic acid) was as follows: 0-40 min 0-100% B → 40-45 min 100% B.

Using HPLC-PDA and LC/ESI-MS allowed for the identification of the major secondary metabolites of hydroalcoholic extract of the *C. brasiliense* leaves. Chemical identification was performed based on aspects of chemosystematics, UV spectra and comparison of retention time. The identifications were confirmed using Database ChemSpider^®^, and literature search using the SciFinder Scholar^®^ tools.

### Statistical analysis

Data normality was evaluated by using the Shapiro-Wilk test. The Poisson regression test (STATA version 14) was used for comparing the counts of larvae, pupae, and adults from different groups (per analysis point). Calculations of IE_50_ for *A. aegypti* and LC_50_ for *D. rerio* were done by using probit analysis (GraphPad Prism version 6). Graphs were also constructed using GraphPad Prism. The significance level was set at 5%.

## RESULTS

**FIGURE 1:**
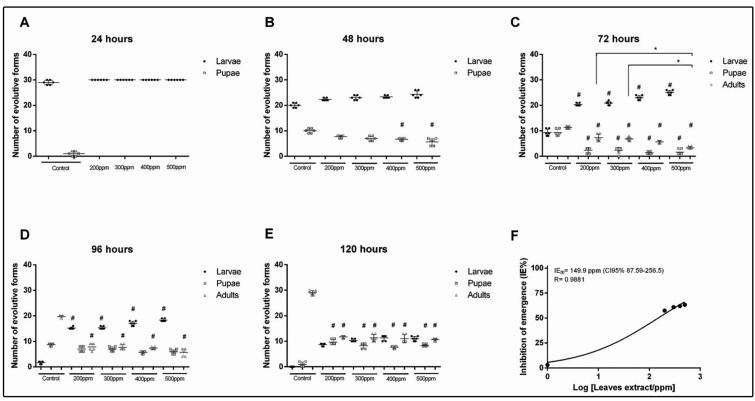
Number of larvae, pupae and, adult *Aedes aegypti* after 120 hours (at 24-hours intervals) with and without contact with different concentrations of the hydroalcoholic extract of *Caryocar brasiliense* leaves **(A-E)** and, the rate of inhibition of adult emergence **(F)**. #Statistically significant differences compared to the control group (with a 5% significance level, and C.I. 95% of estimative that do not include zero). *Statistically significant differences between experimental groups (with a 5% significance level, and C.I. 95% of estimative that do not include zero). **IE**_50_: Emergence inhibition for 50% of the population; **ppm**: parts per million; **CI:** 95% confidence interval; **R:** Linear regression coefficient; **Log:** Logarithm.

No negative effect on larval development was observed in the first 24 hours (Figure 1A). However, after 48 hours, there was a decrease in the number of larvae that successfully developed into pupae at the concentrations 400 ppm (estimate: -0.4; 95% confidence intervals [C.I. 95%]: -0.8 to -0.005; p = 0.047) and 500 ppm (estimate: -0.56; C.I. 95%: -0.98 to -0.14; p = 0.008) compared to the control (Figure 1B). These trends potentiated over time, especially after 72 hours at 500 ppm, when the number of adults was significantly lower compared to that at 200 ppm (estimate: -0.78; C.I. 95%: -1.31 to -0.25; p = 0.003) and 300 ppm (estimate: -0.71; C.I. 95%: -1.25 to -0.18; p = 0.008), besides being lower than in the control group (estimate: -1.22; C.I. 95%: -1.72 to -0.72; p = 0.001) (Figure 1C). As a consequence of this delay in larval development (initiated after 48 hours), there was a decrease in the number of adults that emerged at all concentrations (200-500 ppm) after 96 (Figure 1D) and 120 hours (Figure 1E).

Although no mortality was recorded and adults showed no morphological changes at the end of the experiment, larvae in the test groups that did not develop into adults had a delicate layer of chitin, typical of the early stages of larval development (data not shown).

Growth inhibition was dose-dependent; with increasing concentrations there was a decrease in the number of adults (IE: 200 ppm [57.5%]; 300 ppm [61%]; 400 ppm [62%]; 500 ppm [63.2%]). Therefore, an IE_50_ of approximately 150 ppm was estimated (Figure 1F). 

We also confirmed that the concentration of *C. brasiliense* leaf extract capable of preventing 50% of larvae development was lower than the lethal concentration observed in the *D. rerio* tests, which was 162 ppm (C. I. 95%: 147.7 - 176.4). 

**FIGURE 2:**
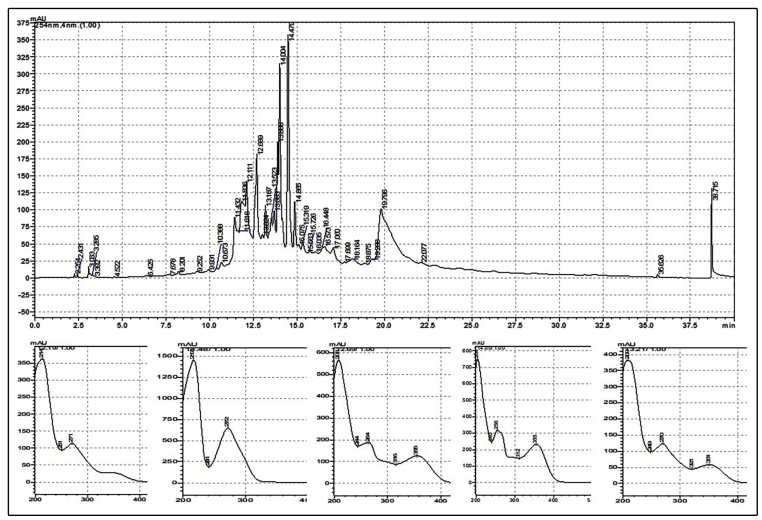
Chromatographic profile and UV bands (254 nm) of the hydroalcoholic extract of *Caryocar brasiliense* leaves. **mAU:** Peak area; **nm**: nanometer.

Analysis of the chromatogram at 254 nm indicated chemical compounds with similar polarities, and retention times with a range of 10.6 min to 19 min. According to the gradient used, these peaks were associated with solvent percentage B ranging from 35.9% to 60% methanol during elution. The UV spectra associated with these peaks showed absorptions at 271 nm and 256-355 nm, characteristic of phenolic substances such flavonoids (Figure 2).

**FIGURE 3:**
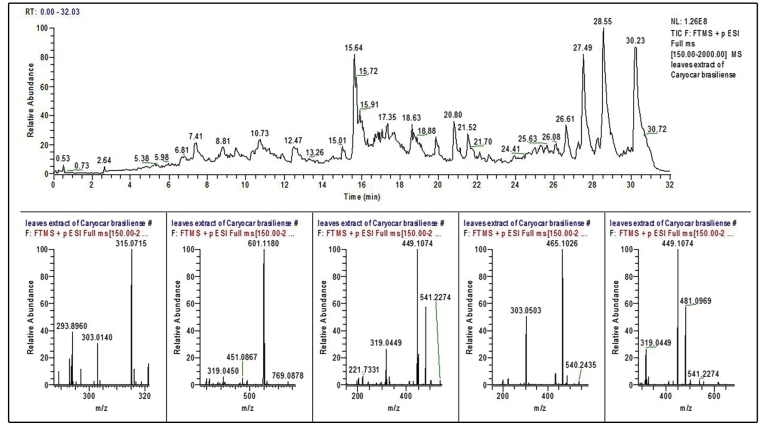
Total ion chromatograms (TICs) of hydroalcoholic extract of *Caryocar brasiliense* leaves. **R**_t_ **:** retention time in minutes; **TIC F:** Total ion chromatograms in position F to expansion of the spectrum of ESI (+) MS FT-ICR of the sample of leaf extract of *C. brasiliense*; ***m/z*:** ratio between area and mass; **NL:** cell volume dimensions.

The total ion chromatograms (TIC) of the hydroalcoholic extract, and the peaks corresponding to the compounds tentatively identified by using LC/ESI-MS are shown in Figure 3 and Table 1. In the same table, we have shown the compounds tentatively identified by using HPLC-DAD and LC/ESI-MS experiments along with their retention times (Rt), detected accurate mass (positive ionization mode) and bibliographic references used for the characterization.

**TABLE 1:** Phytochemical compounds detected and characterized in the hydroalcoholic extract of *Caryocar brasiliense* leaves by using LC/ESI-MS in positive ionization mode.

Peak	R_t_(min)	λ_max_ (nm)	[M+H] ^+^(*m/z*)	Compound	Reference
1	7.41	272	303.0140	Quercetin	Roesler *et al*.^18^
2	8.71	417/470	601.1118	violaxanthin	Azevedo-Meleiro *et al*.^19^
3	8.81	264/358	481.0969	Myricetin3-O-hexoside	Fracassetti *et al*.^20^
4	9.49	264/355	449.1074	Methyl-elagic-3-arabinose acid	Ascari *et al*.^21^
5	10.37	254/355	465.1026	isoquercitrin	Alves *et al*.^22^

**R**_t_ **(min):** retention time in minutes; **λ**_max_ **(nm):** maximum wavelength in nanometer; **[M+H]** ^+^ **(*m/z*):** molecular ion peak protonate; **LC/ESI-MS:** Liquid Chromatography Electrospray Ionization Mass Spectrometry.

## DISCUSSION

Currently, several researchers aim to develop new natural and alternative chemical substances from plant extracts against *A. aegypti* capable of interrupting its life-cycle. Pyriproxyfen (Sumilarv^®^), the main product commercially available for this purpose, was already shown to be ineffective against some *A. aegypti* populations^23^ ^,^ ^24^ *.* The complex chemical composition of natural extracts may reduce the development of resistance by these insects, and exhibit low environmental toxicity due to their biodegradability^25^ ^,^ ^26^, which makes them excellent choices in the search for new insecticidal products.

 The present study showed that L3 larvae of *A. aegypti* failed to develop into mosquitoes when exposed to the hydroalcoholic extract of *C. brasiliense* leaves, although it did not promote larval mortality. The growth regulatory activity of bioproducts can render them as promising insecticides, because affecting the aquatic evolutionary stages may reduce the number of future adults, suggesting that these insects will need a longer time for a new generation to complete their life-cycle^27^.

Based on studies describing the inhibition of emergence rates of adult mosquitoes by plant extracts, the *C. brasiliense* hydroalcoholic extract presents an excellent IE_50_ value when compared with other extracts, such as the methanolic extract of *Tagetes erecta* leaves (IE_50_ = 214.17 ppm), the chloroform extract of *Eclipta prostrata* (IE_50_ = 184.58 ppm) against *Culex tritaeniorhynchus* ^28^, and the aqueous extracts from leaves of *Ricinus communis* against *Anopheles arabiensis* (IE_50_ = 374.97 ppm) and *C. quinquefasciatus* (IE_50_ = 1180.32 ppm)^29^. 

In 2006, the World Health Organization^16^ highlighted the need to conduct toxicological studies for the use of larvicidal products in the environment, including fish and water bugs. Our study showed that the IE_50_ of *C. brasiliense* leaf extract did not affect *D. rerio*, a model for off-target organisms, representing a beneficial environmental aspect of this natural product.

There are many different substances derived from the natural metabolism of plant species, such as saponins, alkaloids, phenolic compounds, terpenoids, or flavonoids, which may affect the development of these mosquitoes individually or synergistically^27^ ^,^ ^28^ ^,^ ^30^ ^,^ ^31^. One of the main mechanisms of action of these substances could possibly involve an antioxidant activity interfering with the morphology and physiology of the larvae, inducing a lethargic behavior by affecting the nervous system of these insects^32^. Specifically, for *A. aegypti*, some authors have described that the *Azadirachta indica* leaf extract contains a substance called azadiractin, which shares structural similarities to ecdisone, and is associated with an inhibition of larval growth, most likely by blocking the production of substances located in their central nervous system, interfering with chitin formation^33^ ^,^ ^34^.

Currently, one of the main Insect Growth Regulators (IGRs) used in campaigns against arboviruses, Pyriproxyfen (Sumilarv^®^), is still highly effective against larvae and pupae of *A. aegypti* ^35^. Comparing the results of Pyriproxyfen IE^35^to the data obtained in this study, we observed that the dose of 500 ppm (IE = 63.2%) of *C. brasiliense* leaf extract presented similar results to the dose of 0.006 ppm of Pyriproxyfen (IE = 64.1%). This demonstrates the insecticidal potential of *C. brasiliense* leaf extract as an IGR, considering that there are already reports of resistance of *A. aegypti* to Pyriproxyfen^23^ ^,^ ^24^. Nevertheless, further studies comparing the effectiveness of the hydroalcoholic extract of *C. brasiliense* leaves and Pyriproxyfen still need to be conducted for more accurate conclusions. 

Based on a large body of evidence showing that natural products may present effective substances affecting the development of *A. aegypti* larvae, we verified that the *C. brasiliense* extract exhibited the presence of phenolic substances such as flavonoids, of which we identified five major compounds. 

Among these substances, quercetin is one of the most abundant flavonoids in plants^36^. In the melon fruit fly, *Bactrocera cucurbitae*, quercetin was able to inhibit pupation and the percentage of adult emergence^36^. In *A. aegypti*, a nanosuspension of this substance has been shown to affect the development of the larvae, causing 100% mortality at a concentration of 500 ppm, and the larvae that survived at the lowest concentration were unable to transform into adults^37^. However, data are still controversial because other experiments with *A. aegypti* larvae have failed to show a growth-inhibitory effect, even at high concentrations. However, larval mortality was significantly increased at concentrations of 11, 10 and 7 mg/mL^38^. An important derivative of this substance, isoquercitrin, exhibits anti-tumor effects^39^. However, there is still a lack of knowledge about its insecticidal potential against *A. aegypti*.

Little is known about the pharmacological properties of violaxanthin, however, studies have reported its antioxidant activity^40^. In contrast, benefits from Myricetin3-O-hexoside and methyl-elagic-3-arabinose acid have still not been proven. Thus, it is likely that these substances are not responsible for the inhibition of the emergence of adult *A. aegypti* found in this study. However, the interaction of these molecules with others present in the extract of *C. brasiliense* leaves could have generated a growth-inhibitory effect, but this needs to be further explored. 

In summary, we conclude that the hydroalcoholic extract of *C. brasiliense* leaves has great potential to prevent the development of *A. aegypti* larvae under laboratory conditions, and it exhibited minimal toxicity in our off-target model organism. Additionally, we demonstrated for the first time that the chemical compounds found in *C. brasiliense* leaf extract may also exhibit biological action against *A. aegypti*. However, further tests need to be performed, by using many different solvents and parts of this plant (fruit, root, stem and flowers), in order to obtain the largest possible quantity of secondary metabolites to act against the larvae of *A. aegypti*.
